# ß-Blocker Timolol Prevents Arrhythmogenic Ca^2+^ Release and Normalizes Ca^2+^ and Zn^2+^ Dyshomeostasis in Hyperglycemic Rat Heart

**DOI:** 10.1371/journal.pone.0071014

**Published:** 2013-07-29

**Authors:** Erkan Tuncay, Esma N. Okatan, Guy Vassort, Belma Turan

**Affiliations:** 1 Department of Biophysics, Faculty of Medicine, Ankara University, Ankara, Turkey; 2 INSERM U-1046, CHU Arnaud de Villeneuve, Montpellier, France; Brigham & Women's Hospital – Harvard Medical School, United States of America

## Abstract

Defective cardiac mechanical activity in diabetes results from alterations in intracellular Ca^2+^ handling, in part, due to increased oxidative stress. Beta-blockers demonstrate marked beneficial effects in heart dysfunction with scavenging free radicals and/or acting as an antioxidant. The aim of this study was to address how β-blocker timolol-treatment of diabetic rats exerts cardioprotection. Timolol-treatment (12-week), one-week following diabetes induction, prevented diabetes-induced depressed left ventricular basal contractile activity, prolonged cellular electrical activity, and attenuated the increase in isolated-cardiomyocyte size without hyperglycemic effect. Both *in vivo* and *in vitro* timolol-treatment of diabetic cardiomyocytes prevented the altered kinetic parameters of Ca^2+^ transients and reduced Ca^2+^ loading of sarcoplasmic reticulum (SR), basal intracellular free Ca^2+^ and Zn^2+^ ([Ca^2+^]_i_ and [Zn^2+^]_i_), and spatio-temporal properties of the Ca^2+^ sparks, significantly. Timolol also antagonized hyperphosphorylation of cardiac ryanodine receptor (RyR2), and significantly restored depleted protein levels of both RyR2 and calstabin2. Western blot analysis demonstrated that timolol-treatment also significantly normalized depressed levels of some [Ca^2+^]_i_-handling regulators, such as Na^+^/Ca^2+^ exchanger (NCX) and phospho-phospholamban (pPLN) to PLN ratio. Incubation of diabetic cardiomyocytes with 4-mM glutathione exerted similar beneficial effects on RyR2-macromolecular complex and basal levels of both [Ca^2+^]_i_ and [Zn^2+^]_i_, increased intracellular Zn^2+^ hyperphosphorylated RyR2 in a concentration-dependent manner. Timolol also led to a balanced oxidant/antioxidant level in both heart and circulation and prevented altered cellular redox state of the heart. We thus report, for the first time, that the preventing effect of timolol, directly targeting heart, seems to be associated with a normalization of macromolecular complex of RyR2 and some Ca^2+^ handling regulators, and prevention of Ca^2+^ leak, and thereby normalization of both [Ca^2+^]_i_ and [Zn^2+^]_i_ homeostasis in diabetic rat heart, at least in part by controlling the cellular redox status of hyperglycemic cardiomyocytes.

## Introduction

Diabetes is one of the major risk factors for the development of cardiovascular complications. A specific cardiomyopathy was first recognized by Rubler et al. [1] in diabetic patients with marked mechanical dysfunction [2]. In animal model studies with type 1 diabetic rats, the defects identified in mechanical activity of the hearts include alterations of Ca^2+^ signaling via changes in critical processes that regulate intracellular free Ca^2+^ concentration, [Ca^2+^]_i_ [2], [3], [4], [5]. Recent studies demonstrated that myocardial heart failure is associated with increased oxidative stress and abnormal excitation-contraction coupling (ECC) characterized by depletion of sarcoplasmic reticulum (SR) Ca^2+^ stores and reduction in Ca^2+^-transient amplitude associated with oxidative modification of thiols in both SR Ca^2+^-ATPase (SERCA) and Na^+^/Ca^2+^-exchanger (NCX) [6], [7]. In addition, although there are some contradictions between the experimental results in the literature, it is pointed out that increased oxidative stress, induced by reactive oxygen and nitrogen species (ROS/RNS) derived via hyperglycemia, has important contribution, directly and/or indirectly, to the structural and functional damages in the diabetic cardiomyocytes [8], [9]. In both early and recent studies, depression in contraction and relaxation of cardiomyocytes from streptozotocin (STZ)-induced diabetic rats were found in parallel with reduced rate of rise and decline of intracellular Ca^2+^ transient elicited by electrical stimulation which were mostly attributed to anomalous SERCA and phospholamban (PLN) activities, hyperphosphorylation of SR Ca^2+^ release channel ryanodine receptors (RyR2), and in part to reduced NCX activity [3], [10], [11], [12], [13], [14].

Within the last decade, the role of RyR2 in the mammalian heart has been well-defined. Under physiological conditions, RyR2 controls Ca^2+^ release and regulates cardiac ECC in a manner of macromolecular complex that includes mainly protein kinase A (PKA), FK506 binding protein 12.6 (FKBP12.6 or calstabin2), and Ca^2+^-calmodulin kinase II (CaMKII). In heart failure, PKA-mediated hyperphosphorylation of RyR2 causes dissociation of calstabin2 from RyR2 which results in an abnormal Ca^2+^ leak through RyR2 leading to cardiac dysfunction [3], [15], [16], [17]. We and others showed that ventricular myocytes isolated from STZ-induced diabetic rat hearts exhibit increased frequency of spontaneous Ca^2+^ sparks leading to an increased basal [Ca^2+^]_i_ which are related, in part, with a leaky SR associated with hyperphosphorylation of RyR2 and decreased protein level of FKBP12.6 in 4-week diabetic rat cardiomyocytes [3].

It has been also shown that intracellular basal free Zn^2+^ level (resting free Zn^2+^) can increase rapidly in cardiomyocytes due to Zn^2+^ release from intracellular stores by reactive, ROS/RNS [18]. Moreover, ROS/RNS have been proposed to contribute to direct and/or indirect damage to cardiomyocytes in diabetes [19], [20], providing a close relationship between both increased and deleterious effects of intracellular basal free Zn^2+^ level in heart.

Hyperadrenergic state is an important marker for increased risk of mortality in patients with heart failure, and therefore it is a logical basis as to why treatment with β-blockers reduces the rate of mortality of these patients, as well as restoring cardiac function in experimental animal studies [21], [22]. It is now well-documented that ligand-binding to β-adrenoreceptors (β-ARs) activates adenylyl cyclase via G-proteins, resulting in a marked elevation in cAMP and PKA activation. Indeed, β-ARs are downregulated in the diseased heart, and uncoupled from downstream signaling via G proteins [23]. Moreover, an early study by Feldman et al. [23] showed a relation between decreased cAMP production and thus PKA activity and contractile dysfunction in failing hearts, while a few studies examined the PKA phosphorylation–dependent RyR2 dysfunction in similar failing hearts [24].

Several studies have investigated the preventive role of various β-blockers on the development of heart failure by restoring RyR2 dysfunction [25], [26]. Furthermore, it was mentioned that non- selective β-blockers, aside from their β-blockage action [27], [28], [29], exert adrenoceptor-independent effects including scavenging of free radicals leading to controlled cellular redox status [30], [31]. We reported that chronic treatment with either timolol or propranolol demonstrates beneficial effects on heart function in male rats during increasing-age, whereas timolol, but not propranolol exerts similar beneficial effects in female rats [32], [33]. Additionally, our recently published data has demonstrated that chronic treatment with propranolol seems to prevent diabetes-related changes in heart function by controlling intracellular Ca^2+^ signaling and preventing the development of left ventricular remodeling in diabetic cardiomyopathy without any antioxidant action as systemic or targeting heart of STZ-rats [32]. Furthermore, in a cell culture study, Miyamoto et al. [34] showed that both nipradilol and timolol are potent protective agents against increased oxidative stress. Also, most important data related with direct ROS scavenging action of timolol are presented by [35] and [36] via a nice comparison with other β-blockers. Moreover, we showed previously a profound cardioprotection with timolol in a female rat model of aging-related altered left ventricular function via prevention of the antioxidant system dysfunction, including increased lipid peroxidation, decreased ratio of reduced glutathione to oxidized glutathione, and decreased activities of thioredoxin reductase and glucose-6-phosphate dehydrogenase of left ventricular heart samples [32]. All above data can provoke ones to investigate whether or not timolol action is different among the other known β-blockers in diabetic subjects.

Taken into consideration the preventive role of β-blockers on the structure and function of RyR2 macromolecular complex in various studies on both human and animal heart failure models, we aimed to investigate the mechanisms that underlie the benefits observed with β-blocker timolol treatment on diabetic cardiomyopathy. Our data showed that a long-term treatment of diabetic male rats with a β-AR blocker timolol prevented diabetes-induced depressed basal activity of the left ventricle. Moreover, we report for the first time that this important preventive effect of timolol is associated with its normalization action on the RyR2 macromolecular complex, balanced intracellular Ca^2+^ and Zn^2+^ homeostasis, and a balanced level of oxidant to antioxidant ratio in both heart and circulation. We also report that timolol-treatment significantly normalized depressed levels of some Ca^2+^ handling regulators, such as NCX and phospho-PLN to PLN ratio. These observations may account for some of the beneficial effects of β-ARs blockage with timolol in diabetic cardiomyopathy confirmed by severe heart dysfunction.

## Materials and Methods

### Induction of diabetes

Diabetes was induced by a single injection of streptozotocin (STZ, 50 mg/kg dissolved in 0.1 M citrate buffer at pH 4.5; intraperitoneal; Sigma-Aldrich) in 3-month-old male Wistar rats (weighing 200–250 g) following an overnight fast as described previously [9]. Blood glucose concentration >3-fold that of the age-matched control at both 7 days and 12 weeks post-STZ injection was the criterion for experimental diabetes. One group of diabetic rats was treated with timolol (5 mg/kg daily) for 12 weeks. All animals were handled in accordance with the Guide for the Care and Use of Laboratory Animals published by the US National Institutes of Health (NIH publication No. 85–23, revised 1996). The protocol is approved by the Committee on the Local Ethics of Animal Experiments of Ankara University (N°: 2007-11-38).

### Langendorff-perfused cardiac function

The left ventricular developed pressure (LVDP), left ventricular end diastolic pressure (LVEDP) and the rates of changes in developed pressure (±dP/dt) of isolated hearts were measured as previously described [37].

### Action potential recording

Papillary muscle strips isolated from left ventricle were used to monitor intracellular action potentials using a conventional glass microelectrode connected to a preamplifier as described previously [9]. Action potential parameters such as amplitude of action potential, membrane potential, and repolarisation phases at 75% and 90% (APD_75, 90_) were determined and compared between groups.

### Cell isolation

Cardiomyocytes from rat hearts were isolated freshly as described previously [3]. The cells were suspended in HEPES buffer with 1 mM Ca^2+^ and 0.5% bovine serum albumin (pH 7.4) and were kept at 37°C prior to being used for the experiments.

### Patch-clamp experiments

Whole-cell patch-clamp recordings were performed as described previously [3]. Briefly, L-type Ca^2+^ currents (I_Ca_) were recorded in cardiomyocytes isolated from the left ventricle of the hearts at room temperature (22±2°C) in the presence of cesium to inhibit K^+^ currents. Na^+^ current was inhibited by a voltage ramp protocol from a holding potential of –80 mV to –50 mV. Patch pipettes (1.0–1.2 MΩ) were filled with a solution (in mM): 100 CsCl, 20 TEA-Cl, 10 EGTA, 5.4 ATP-Na_2_ and 10 HEPES; pH 7.3. Cells were held at –80 mV and the current amplitude was estimated as the difference between peak inward current and the current level at the end of the 200 ms pulse.

### Global and local cytosolic Ca^2+^ measurements

Freshly isolated left ventricular myocytes were loaded with the fluorescent Ca^2+^ indicators either Fura-2 (4-µM Fura-2 AM) or Fluo-3 (2-µM Fluo-3 AM). Transient intracellular free Ca^2+^ concentration ([Ca^2+^]_i_) changes under electric-field stimulation (Ca^2+^ transients as fluorescence ratio changes) were measured from Fura-2 loaded cardiomyocytes at room temperature (21±2°C) as described previously [3]. The fluorescence ratio F_340/380_ of the emitted light (with a frequency of 10 Hz) on excitation at 340 and 380 nm was used as an indicator of intracellular free Ca^2+^ changes. Peak amplitude (difference between basal and peak F_340/380_ ratios; ΔF) time to peak fluorescence (TP) and half-decay time of fluorescence (DT_50_) shifts between groups were estimated by a trend fit to whole Ca^2+^ transients evoked by field stimulation.

Tiny local Ca^2+^ releases in quiescent cells, Ca^2+^-sparks were measured from Fluo-3 loaded freshly isolated cardiomyocytes as described previously [3]. Briefly, an X60 (numerical aperture of 1.2) water immersion objective was used for imaging cardiomyocytes (Leica, SP5). The 488 nm argon laser line was used to excite Fluo-3, and the emitted fluorescence was collected with a long-pass filter set at 505 nm. The parameters of fluorescence changes such as peak amplitude (ΔF/F_0_, where ΔF = F−F_0_; F was identified as local maximum elevation of fluorescence intensity over basal level, F_0_), TP, spatial width (full width at half-maximum intensity, FWHM), and full duration half maximum (FDHM) were calculated automatically by using ImageJ program.

In another group of experiments, we repeated the experiments in diabetic cardiomyocytes incubated with either timolol (10-μM) or glutathione (4-mM) for 1 hour at 37°C.

### Measurement of resting levels of cytosolic Ca^2+^ and Zn^2+^

To monitor the resting (or basal) level of intracellular free Zn^2+^ as well as Ca^2+^ in quiescent cells, we used two different Zn^2+^ sensitive fluorescence dye-loaded cells, by using either a ratiometric Fura-2 (4-µM Fura-2 AM) for a PTI Ratiomaster microspectrofluometer (working with FELIX software; Photon Technology International) or a non-ratiometric FluoZin-3 (3-μM FluoZin-3 AM) for fluorescence spectrometer (Jasco FP-6500, Japan).

We utilized an indirect approach to monitoring intracellular free Zn^2+^ level in freshly isolated cardiomyocytes by which the fluorescence attributable to Zn^2+^ was quenched by chelating the free intracellular Zn^2+^ with the heavy metal chelator N,N,N’,N’-tetrakis(2-pyridylmethyl) ethylenediamine (TPEN) similiar to early studies by us and others [18], [38], [39], [40]. Our approaches were based on the assumption that chelation of Zn^2+^ by TPEN did not affect intracellular free Ca^2+^ or other heavy metal ions due to fact that TPEN has very high affinities for heavy metals, for example, K_a_ = 10^10.27^ M^−1^, 10^14.61^ M^−1^, and 10^15.58^ M^−1^ for Mn^2+^, Fe^2+^, and Zn^2+^, respectively [41], [42]. In addition, the dissociation constant for the complex Ca-TPEN is ∼40 µM, compared with 10^−15.6^ M for Zn-TPEN [41], indicating that only negligible amounts of intracellular Ca^2+^ would be bound to TPEN under normal conditions. We first performed in vitro calibrations of Fura-2 with Zn^2+^ and Ca^2+^ in a manner similar to those described at 37°C in a manner similar to those described previously [18], [38], using various Zn^2+^- and Ca^2+^-buffered solutions with EGTA and nitrilotriacetic acid, and the equation given in our previous study is used to fit the data considered competitive binding between Zn^2+^ and Ca^2+^ for Fura-2 [18], [38]. The R_min_, R_max,Zn_, and R_max,Ca_ were determined directly from Fura-2 loaded cardiomyocytes exposed to calibration solutions supplemented with 1 µM digitonin to hyperpermeabilize the cell membrane. In here, we did not aim to quantitate either the Zn^2+^ or Ca^2+^, we just presented fluorescence ratio changes for both Zn^2+^ or Ca^2+^, and results are presented as % of the control.

To monitor the basal levels of intracellular free Zn^2+^ and Ca^2+^, we repeated the above measurements in FluoZin-3AM loaded quiescent cells by using a fluorescence spectrometer (Jasco FP-6500, Japan). FluoZin-3 is highly selective for Zn^2+^ [43] and is not perturbed by Ca^2+^ or Mg^2+^ [40]. The fluorescence level corresponding to cytosolic free Zn^2+^ is calculated by using [Zn^2+^]_i_  =  K_d_X(F-F_min_) /(F_max_-F) formula, where the K_d_ for FluoZin-3 is 15 nM. F_max_ and F_min_ represent the maximum fluorescence intensity (with 10 µM a zinc-ionophore of 1-hydroxypyridine-2-thione, ZnPT) and the minimum fluorescence intensity (with 50 µM TPEN) while F represents the measured basal intensity. The intrinsic ﬂuorescence measurements were performed on ﬂuorescence spectrophotometer (Jasco FP-6500, JAPAN) with a Cuvette and temperature-controlled water bath. Same amount of cardiomyocytes (∼3000 cells/cuvette) for every experiments, placed into a cuvette which is stirred up with a small magnet for measuring the basal level of Zn^2+^ by spectrophotometer. Florescence intensities were acquired at 1 Hz, 490-nm excitation wavelength and collected at 525-nm. The fluorescence emissions were derived after subtracting of auto-florescent of the cells. Results are presented as % of control.

Although most of the published data demonstrated that both Fura-2 and FluoZin-3 have very high sensitivity to free Zn^2+^ rather than the other heavy metal ions [18], [38], [39], [43] while a metal-chelator TPEN has very high affinity to Zn^2+^ [40], [41], [42], yet there is limitation to monitor intracellular free Zn^2+^ and it is not possible to exclude even any little contribution of other ions, including Ca^2+^ or Mg^2+^.

To asses SR Ca^2+^ load experiment, caffeine-induced Ca^2+^ transients were induced in Fura-2 loaded cardiomyocytes by a rapid application of 10-mM caffeine as described previously [3].

### Western blot analysis

For preparation of tissue homogenates, frozen heart samples from left ventricle were crushed at liquid N_2_ temperature and then homogenized to measure the phosphorylation and protein levels of contractile machinery complex as described previously [3] (CaMKII, phospho-CaMKII-Thr286, PKA, phospho-PKA-Thr198, FKBP12.6, RyR2, phospho-RyR2-Ser^2808^, Na/Ca-exchanger (NCX1), sarcoplasmic reticulum Ca-ATPase (SERCA2), phospholamban (PLN), phospho-PLN-Thr17, and β-actin were identified using specific antibodies with recommended dilutions from companies of either Santa Cruz biotechnology, USA or Badrilla Ltd., UK. Density analysis of protein bands was performed using Image J programme.

The similar Western blot analysis was also performed in the diabetic rat cardiomyocytes from left ventricle incubated with either timolol (10-μM) or zinc-ionophore of 1-hydroxypyridine-2-thione (ZnPT) (1 or 10- μM) for 20–30 min at 37°C.

### Determination of protein-thiols

Oxidized and free levels of protein-thiols (protein-SH) were measured in heart homogenates as described previously [20].

### Measurement of β-adrenergic receptor density

Density of total β-adrenergic receptors in crude membrane preparations from ventricular parts of the hearts were measured as described previously [28]. Briefly, a single concentration of ^125^I-cyanopindolol was used at a concentration of 800 pmol/L (80 times of its K_d_ value) to saturate β-AR. Nonspecific binding was determined in the presence of 100 µmol/L of unlabeled timolol and specific binding was calculated as the difference between total and nonspecific bindings.

### Measurement of total oxidant and antioxidant status

Total oxidant status (TOS) and total antioxidant status (TAS) measurements were performed in both the left ventricular heart homogenates and plasma by using commercial kits (Rel Assay Diagnostics). TOS measurement was based on the oxidation of the ferrous ion–*o*-dianisidine complex to ferric ion by the oxidants present in the sample. The assay was calibrated with H_2_O_2_ and the results are expressed in terms of μmol H_2_O_2_ equivalent per liter.

For the TAS assay, the reduced 2,2′-azino-bis (3-ethylbenz-thiazo-line-6-sulfonic acid) (ABTS) molecule was oxidized to ABTS.^+^ by using hydrogen peroxide alone in acidic medium (the acetate buffer 30 mmol/L, pH 3.6). Antioxidants present in the sample accelerate the bleaching rate to a degree proportional to their concentrations. This reaction can be monitored spectrophotometrically and the bleaching rate is inversely related with the total-antioxidant status (TAS) of the sample. The reaction rate is calibrated with Trolox standard (an analog of vitamin E) and is expressed as mmol Trolox equivalent per liter.

### Direct measurements of timolol antioxidant activity and antioxidant status

Using the same principle and method mentioned in the previous section, the assay was calibrated with hydrogen peroxide (H_2_O_2_) and the results are expressed in terms of H_2_O_2_ equivalent per liter (μmol H_2_O_2_ Equiv./L). We used increasing concentrations of timolol (in μM; 10-30-100-300) and measured the absorbance changes (at 530 nm) of H_2_O_2_ induced signal and evaluated the data as antioxidant activity.

For the second part of measurement, the assay was calibrated with a stable antioxidant standard solution which is traditionally named as Trolox Equivalent. The change of absorbance at 660 nm is related with total antioxidant level of the samples (increasing timolol concentrations in μM; 10-30-100-300). We measured the capacity of the samples to reduce chromogen ABTS and evaluated the data as antioxidant status.

### Data analysis

Groups were tested and compared using one-way ANOVA and Tukey post hoc test. Values of p<0.05 were taken as statistically significant. Significant levels are given in the text, and data are presented as mean ± SEM.

## Results

### General effects of 12-week timolol-treatment of diabetic rats

**Figure 1:**
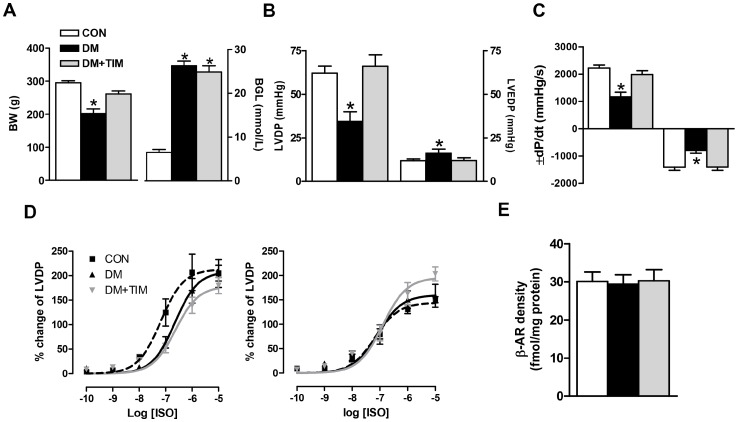
Timolol treatment prevents cardiac dysfunction via normalizing basal mechanical activity. (A) Body weights (left) and blood glucose levels (right) of the rats at the end of the 12-week experimental period. Left ventricular developed pressure, LVDP (B, left) and left ventricular end diastolic pressure, LVEDP (B, right), the rates of changes in the developed pressure (±dP/dt) (C). Bar graphs in A to C represent mean ± SEM values from control, diabetic, and timolol-treated diabetic groups (number of rats in the groups; n_CON_ = 18, n_DM_ = 24, and n_DM+TIM_ = 18, respectively) Effects of submaximal concentrations of a non-specific agonist, isoproterenol (ISO) on LVDP responses measured in short-term (left) and long-term (right) timolol-treated as well as untreated diabetic rats (D). Concentration–response curves for ISO represent the inotropic responses as % of their initial values and the LogEC_50_ value, which is equal to the concentration required to produce 50% of the maximal response induced by the agonist as determined from log-probit plots of individual response vs. concentration. LogEC_50_ values for ISO responses: −7.2±0.2, −6.6±0.2, −6.6±0.1 for short-term (4-week) protocol groups and −7.2±0.1, −7.1±1.2, −6.9±0.1 for long-term (12-week) protocol groups, in CON (n = 7/8 for 4-week/12-week), DM (n = 6/7 for 4-week/12-week), and DM+TIM (n = 7/7 for 4-week/12-week), respectively. The total β-AR density was measured in the crude membrane preparation from the hearts of long-term protocol groups by using saturation-binding technique with ^125^I-cyanopindolol (E). Bar graphs and the data points in each curve represent mean ± SEM values from CON (n = 6), DM (n = 7), DM+TIM (n = 6) groups. Significant at ^*^p<0.05 *vs*. CON.

As a typical model of type 1 diabetes, STZ-injected rats displayed hyperglycemia as indicated by significant increases in water intake and plasma glucose level compared with age-matched controls starting from week 1 until week 13 after STZ-injection. Timolol-treatment had no significant effect on plasma glucose level; however, it improved diabetic symptoms by significantly reducing weight loss although the weight of the subjects remained less than that of the controls (Fig. 1A). Cardiac hypertrophy that occurred during diabetes was also prevented by timolol. The mean cell capacitance of the untreated diabetic group was significantly higher (229.7±9.8 pF, n_cell_ = 26 and n_rat_ = 6) than that of the control (193.5±11.3 pF, n_cell_ = 20 and n_rat_ = 6), but it was not significantly different in the timolol-treated diabetics compared to the controls (181.7±6.5 pF, n_cell_ = 34 and n_rat_ = 7).

Cardiovascular function was evaluated by left ventricular (LV) hemodynamic analysis. For comparison as preliminary data, we used either short-term (4-week) or long-term (12-week) timolol-treatment. The basal contractile activity of the heart (left ventricular developed pressure, LVDP) from either 4-week or 12-week diabetic rats was found to be depressed compared to that of the age-matched controls. Timolol-treatment of diabetic rats either short-term or long-term exerted a significant preventive effect on the depressed basal contractile activity of the heart. The LVDP and end diastolic pressure, LVEDP, and maximum rate of rise/fall of LVDP (±dP/dt) in 12-week timolol-treated or untreated diabetic rat heart are given in Fig. 1B and C. Timolol-treatment either short-term or long-term of diabetic rats exerted similar but significant preventive effect on the depressed basal contractile activity of the heart.

We previously reported a significant decrease in the potency of isoproterenol (ISO) on LVDP in 4-week diabetic male rats without a significant change in the maximum response [44]. As can be seen from Fig. 1D (left), a 4-week timolol-treatment of diabetic rats did not affect the ISO responses measured with LVDP changes. In a 12-week timolol-treatment of diabetic rats, the maximum ISO response measured with 10^−5^ M was found to be slightly but significantly increased (without any effect in LogEC_50_) in the treated diabetic group compared with those of both untreated diabetic and age-matched control groups (Fig. 1D, right). Besides, β-ARs densities were similar in myocardial membranes from control and diabetic rats and not modified after chronic timolol-treatment (Fig. 1E).

### Timolol-treatment prevents changes in cardiac electrical activity of the diabetic rat heart

**Figure 2:**
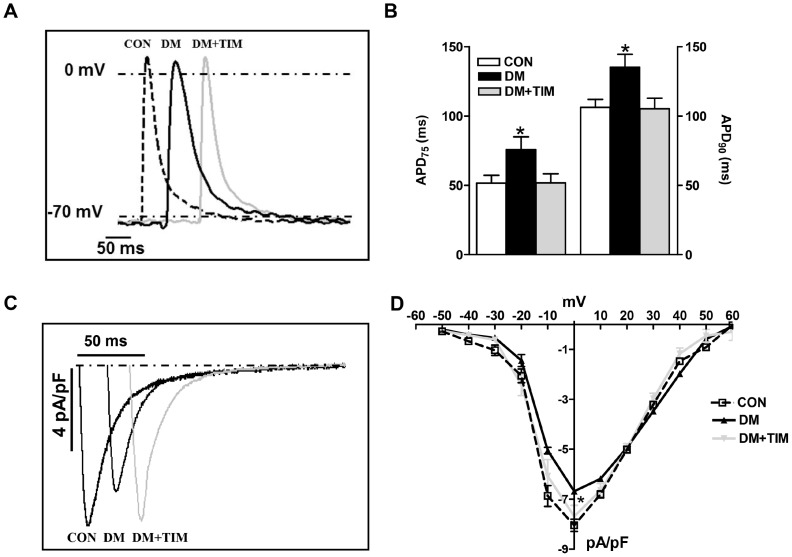
Timolol treatment restores prolonged action potential duration in the papillary muscle strips of the left ventricle and the depressed L-type Ca^2+^-channel currents in the ventricular cardiomyocytes from the diabetic rat heart. (A) Representative intracellular action potential recordings in papillary muscle strips from left ventricle (inset). In here, traces are shifted for sake of clarify. (B) The mean changes in the action potential duration (as % repolarization at 75, 90; APD_75,90_) are presented as bar graphs (represents mean ± SEM from control, CON with white bars; from 8 rats, diabetic, DM with black bars; from 7 rats, and timolol treated diabetic rats, DM+TIM with gray bars; from 8 rats). (C) Representative L-type Ca^2+^-channel currents recorded at 0 mV depolarization (inset; time shifted for clarity), and (D) current–voltage relationship as current density (calculated by dividing their amplitude to their cell capacitance) in freshly isolated cardiomyocytes from control, diabetic and timolol-treated diabetic rats (CON; n = 20 cells from 6 rats, DM; n = 26 cells from 6 rats, and DM+TIM; n = 34 cells from 7 rats). Currents were recorded at room temperature (22±2°C) in the presence of cesium to inhibit K^+^ currents and Na^+^ current was inhibited after a voltage ramp protocol from a holding potential of –80 mV to –50 mV. Voltage pulses were applied from a holding potential of −80 mV to between −60 mV and +60 mV, with 10 mV voltage-steps. Data points on the curves represent mean ± SEM values. Significant at ^*^p<0.05 *vs*. CON.

Diabetes significantly prolonged the action potential duration (APD), mainly the repolarizing phase at 75% and 90% repolarization (APD_75_ and APD_90_) in isolated left ventricular papillary muscles (Fig. 2A and B). Timolol-treatment significantly prevented the prolongation in action potential observed in the untreated diabetic rat heart. The resting membrane potential of 6-month-old diabetic rats (−70.5±0.7 mV) was significantly more depolarized than age-matched controls (−74.4±1.3 mV). Timolol-treatment of the diabetic rats maintained the resting membrane potential (−73.8±0.9 mV).

To determine whether a change in L-type Ca^2+^ -currents (I_CaL_) contributes to these changes in the electrical activity, I_CaL_ was examined by using the whole cell patch-clamp technique. In contrast to our previous study with 4-week-old diabetic rats [3], I_CaL_ densities recorded at various membrane potentials were significantly reduced in cardiomyocytes from 12-week diabetic rats when compared to that of age-matched controls (Fig. 2C and D). I_CaL_ densities at 0 mV were −6.5±0.5 pA/pF and −8.1±0.5 pA/pF in the untreated diabetic and control groups, respectively while the time courses of these currents were similar among the groups (inactivation time course of I_CaL_ was calculated by fitting the curve traces by two exponential equations as described previously, [45] (data not given). Timolol-treatment significantly prevented I_CaL_ decrease (−7.8±0.4 pA/pF) without any effect on the time course of the currents. Although slower time constants were generally associated with smaller I_CaL_ densities, we have similar time courses with smaller current densities in the diabetic group. In addition, we calculated the quantity of total electrical charges carried by I_CaL_ over a 200 ms depolarizing period at 0 mV. The total electrical charges carried by I_CaL_ at 0 mV in the untreated diabetic group was significantly less (3.0±0.7 amol/pF) than in the control group (4.0±0.7 amol/pF), while this decrease in the diabetic group was partially but significantly prevented with timolol-treatment (3.5±0.6 amol/pF).

### Timolol regulation of intracellular global Ca^2+^ changes

**Figure 3:**
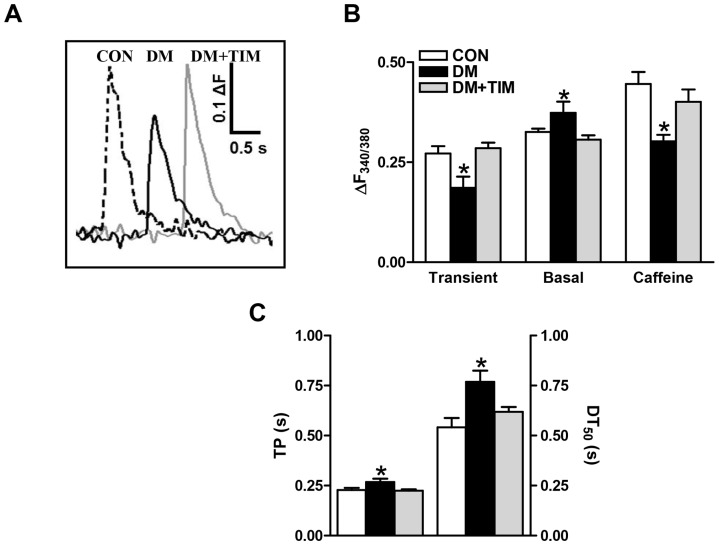
Effect of timolol treatment of diabetic rats on intracellular global Ca^2+^ changes. (A) Representative Ca^2+^ transients in freshly isolated cardiomyocytes loaded with Fura-2 and field-stimulated at 0.2 Hz, (B) changes in peak amplitude of the fluorescence related with global Ca^2+^ transients (ΔF_340/380_ = F_340/380_Peak-F_340/380_Basal) (left), basal level of intracellular Ca^2+^ (middle), and caffeine-induced peak Ca^2+^ transients elicited in the cardiomyocytes (right), and the effect of timolol-treatment of diabetic rats (DM+TIM) on the time course (time to peak fluorescence, TP and half-decay time of fluorescence (left), DT_50_ shifts between groups were estimated by a trend-fitting to whole Ca^2+^ transients evoked by field stimulation of intracellular global Ca^2+^ changes (right) recorded from freshly isolated ventricular cardiomyocytes (C). Bar graphs represents mean ± SEM of 12–17 cells from at least 5 animals for each group protocol. Significant at ^*^p<0.05 *vs*. CON.

To further understand the restoring effects of timolol on diabetic rats, we first performed some additional experiments to monitor [Ca^2+^]_i_ transients elicited by electrical-field stimulation of cardiomyocytes. In line with our previous observations on 5-week STZ-induced diabetic cells [3], the mean peak amplitude of ΔF_340/380_ was significantly smaller in 12-week diabetics than in the control cells (Fig. 3A and B). Furthermore, time-to-peak amplitude (TP) and half-time for recovery (DT_50_) of the Ca^2+^ transients recorded from untreated diabetic rat cells were significantly prolonged compared with that of the control (Fig. 3C). Timolol-treatment of STZ-diabetic rats for 12-week prevented markedly both the depression in the fluorescence intensity and the prolongation in the time course of the intracellular Ca^2+^ transients (Fig. 3A and B). The averaged diastolic value of ΔF_340/380_ (basal level of intracellular free Ca^2+^) in cardiomyocytes from untreated diabetic rat heart was found to be significantly higher than that of age-matched control, and timolol-treatment prevented the increase in basal level of intracellular free Ca^2+^ (Fig. 3B; middle and left bars).

Caffeine application on cardiomyocytes caused a sudden and transient increase in the intracellular free Ca^2+^ due to Ca^2+^ release from the sarcoplasmic reticulum, SR. The size of the caffeine-induced Ca^2+^ transient has been used to assess the SR-Ca^2+^ load of cardiomyocytes from control and diabetic rats as well as timolol-treated diabetic rats. To assure stable SR-Ca^2+^ load, cells were first stimulated, then caffeine (10-mM) was rapidly applied 30 s after cessation of electrical stimulation. The caffeine-induced Ca^2+^ transient recorded in cardiomyocytes from diabetic rats was smaller than that of the control one, while the caffeine-induced Ca^2+^ transient was not significantly different in timolol-treated diabetics compared to that of the control (Fig. 3B; right bars), implying that timolol-treatment maintained SR-Ca^2+^ load.

### Timolol regulation of intracellular basal free Zn^2+^ level

**Figure 4:**
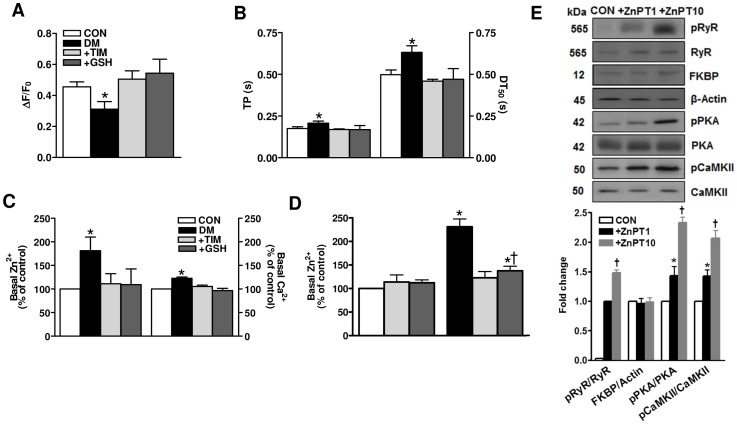
Acute effect of timolol on intracellular global Ca^2+^ changes, and the basal levels of intracellular both free Zn^2+^ and Ca^2+^ in ventricular hyperglycemic cardiomyocytes. (A) Incubation of diabetic cardiomyocytes with either 10-μM TIM or, for comparison 4-mM GSH for 1 hour enhanced the intensity of intracellular free Ca^2+^ transients under electric-field stimulation at 0.2 Hz (left) and shortened their time courses as well, significantly (right) (B). Diabetic cardiomyocytes were incubated for 1 hour with either 10-μM TIM or 4-mM GSH. (C) To record basal levels of both intracellular free Zn^2+^ and Ca^2+^ in resting cells in parallel, we used a ratiometric Fura-2 (4-µM Fura-2 AM) loaded cells and 50-µM N,N,N′,N′-tetrakis (2-pyridylmethyl) ethylenediamine (TPEN) (for more information about methods, see [28]). (D) FluoZin-3 (3-μM FluoZin-3 AM) loaded cells used to measure basal level of intracellular free Zn^2+^. Incubation of cardiomyocytes isolated from either normal or diabetic rat hearts with either TIM or GSH did not affect FluoZin-3 intensity related with basal level of intracellular free Zn^2+^, while these both incubations induced significant decreases the increased FluoZin-3 intensity in the diabetics. (E) Incubation of cardiomyocytes with a zinc-ionophore of 1-hydroxypyridine-2-thione (ZnPT; 1-μM and 10-μM) at 37°C for 20–30 min did not affect total protein levels of RyR2 and its accessory protein of RyR2 macromolecular complex FKBP12.6 and two important kinases PKA and CaMKII while phosphorylation levels of RyR2 as well as PKA and CaMKII (pPKA and pCaMKII, respectively) increased markedly with ZnPT incubation in a concentration-dependent manner. Values for controls (CON; n_rat_ = 5, n_cell_ = 25), diabetics (DM; n_rat_ = 5, n_cell_ = 30), TIM- or GSH-incubated (+TIM or +GSH) diabetics (n_rat_ = 6, n_cell_ = 24 or n_cell_ = 22), and ZnPT incubated controls (n_rat_ = 5, n_cell_ = 30 for each protocol) are expressed as mean ± SEM. Significant at ^*^p<0.05 *vs*. CON.

Under both *in vitro* and *in vivo* conditions, exogenously enhanced oxidative stress or diabetes-induced oxidative stress is known to increase the basal levels of intracellular free Ca^2+^ and, as well, Zn^2+^ which is a strong indicator of the cellular level of oxidative stress in cardiomyocytes [18], [20]. For an assessment of possible antioxidant effects of timolol, first we compared the changes induced by either timolol or reduced glutathione, GSH treatment of cardiomyocytes from diabetic rats compared to age-matched controls. Incubation of diabetic cardiomyocytes with either 10-μM timolol, or with 4-mM GSH for 1 hour enhanced the intensity of fluorescence related with intracellular free Ca^2+^ transients under electric-field stimulation, and also significantly shortened their time courses (Fig. 4A and B). In addition, incubation for 1 hour of diabetic cardiomyocytes with either 10-μM timolol or with 4-mM GSH markedly prevented the increases in the basal levels of intracellular free Zn^2+^ and Ca^2+^ (by using either Fura-2AM or FluoZin-3AM), while these compounds did not affect the basal Zn^2+^ level in control cells (Fig. 4C and D, respectively).

Zinc ion is essential for numerous cellular functions although it is toxic for live cells [46]. Zinc ion homeostasis is therefore dynamically maintained by a variety of transporters, stores, and other proteins distributed in distinct cellular compartments. For assessment of the possibility of increased intracellular basal Zn^2+^-induced RyR2 hyperphosphorylation, which may in turn contribute to the cardiac dysfunction in diabetic subjects, we measured the phosphorylation levels of RyR2 with respect to its protein level with a zinc-ionophore of 1-hydroxypyridine-2-thione (ZnPT) under *in vitro* conditions. As can be seen from Fig. 4E, total protein levels of both RyR2 and accessory protein of RyR2 macromolecular complex, FKBP12.6 were not affected with either 1 µM or 10 µM ZnPT incubation (at 37°C for 20–30 min) of cardiomyocytes freshly isolated from control group rats. However, we measured significantly increased phosphorylation levels of RyR2 with these two ZnPT incubations in a concentration-dependent manner (Fig. 4E). The last data further support the hypothesis that Zn^2+^ disbalance results in a signaling disbalance caused by a local surplus of Zn^2+^ interfering with cellular signaling networks.

It is well-accepted that intracellular free Zn^2+^ plays critical roles in the redox signaling pathway and maintaining the normal structure and physiology of various cell types [18], [38], [47]. In addition, it has been shown that Zn^2+^ has multiple functional effects on Ca^2+^/calmodulin-dependent protein kinase II (CaMKII) [48], [49] while divalent metal ions influence catalysis and active-site accessibility in the cAMP-dependent protein kinase [50]. In order to demonstrate possibility of higher phosphorylation levels of two different kinases (PKA and CaMKII) with increased intracellular Zn^2+^, which are responsible from hyperphosphorylation of RyR2 under pathological conditions, rather than a direct hyperphosphorylated-RyR2 with Zn^2+^, in another set of experiments, we measured the phosphorylation levels of both PKA and CaMKII (with respect to their protein levels) with ZnPT exposures, under *in vitro* conditions. As can be seen from Fig. 4E, their phosphorylation levels are increased with ZnPT in a concentration-dependent manner while their protein levels were not changed with these exposures. Therefore, although it needs further experiments, it is tempting to supeculate a possible pathway for hyperphosphorylated-RyR2 via induction of intracellular free Zn^2+^-dependent PKA-/CaMKII-phosphorylation in our experimental conditions performed in freshly isolated cardiomyocytes.

### Timolol regulation of intracellular local Ca^2+^ changes

**Figure 5:**
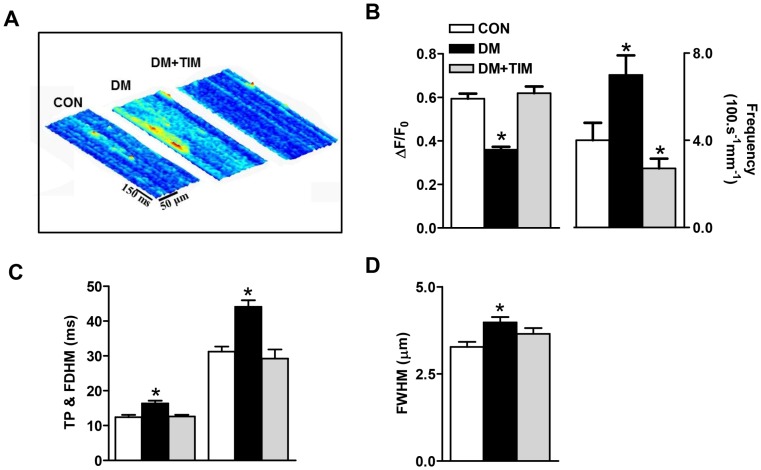
Prevention of increased intracellular basal free Ca^2+^ with timolol in diabetic cardiomyocytes is closely correlated with restoration in Ca^2+^ spark parameters. (A) Representative 3D-reconstruction of a representative line-scan recordings demonstrate the characteristic spreads of fluorescence in space and time in freshly isolated cardiomyocytes from three groups of rats. (B) Bar graphs from control (CON), diabetic (DM), and timolol treated diabetic rats (DM+TIM) are representing peak amplitude (left) and frequency (right), (C) time to peak amplitude TP (left) and full duration at half maximum, FDHM (right), and (D) full width of half maximum of Ca^2+^ sparks (n_rat_ = 5, n_cell_ = 44, n_spark_ = 150; n_rat_ = 5, n_cell_ = 55, n_spark_ = 165; n_rat_ = 5, n_cell_ = 38, n_spark_ = 135 in control CON, diabetic DM, and timolol-treated diabetic DM+TIM groups, respectively). Significant at ^*^p<0.05 *vs*. CON.

The contribution of elementary Ca^2+^ release, Ca^2+^ sparks, to decreased Ca^2+^ content of SR and increased basal Ca^2+^ level was examined in cardiomyocytes isolated from the hearts of a 6-month-old control, a 12-week STZ-induced diabetic, and a 12-week timolol-treated diabetic rats. Representative line-scan images are displayed in Fig. 5. The maximum fluorescence intensity, determined as ΔF/F_0_ at the peak of Ca^2+^ sparks and spontaneous Ca^2+^-spark frequency were calculated from individual gamma distribution function fits (Fig. 5A and B). The two parameters were significantly changed with respect to age-matched controls with a marked reduction in peak Ca^2+^ sparks and a marked increase in their occurrence frequency during diabetes. Time-to-peak (TP), full duration half maximum (FDHM) and full width at half-maximum intensity (FWHM),of the peak Ca^2+^ sparks were also found to be significantly enhanced in untreated diabetics (Fig. 5C and D, respectively). Timolol-treatment of the diabetic rats significantly prevented diabetes-induced changes in all Ca^2+^ sparks parameters.

**Figure 6:**
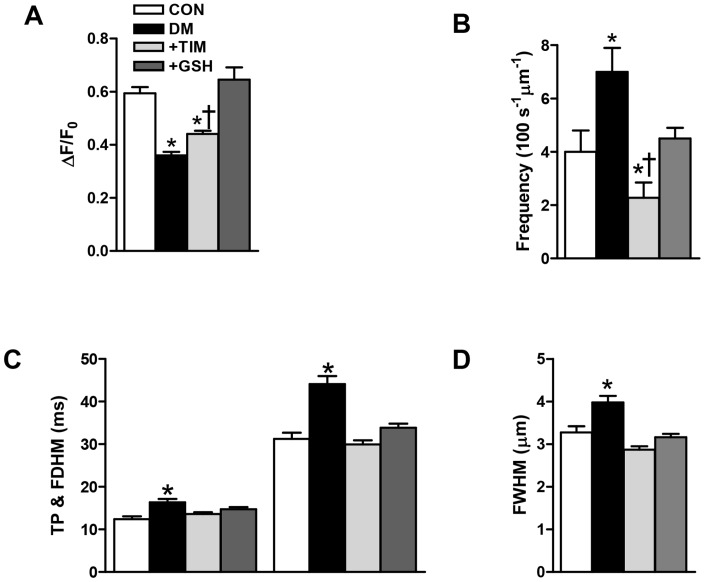
*In vitro* experiments with either timolol or glutathione confirmed the cardioprotective effects of timolol via prevention of increased basal free Ca^2+^ in diabetic cardiomyocytes due to a restoration in Ca^2+^ spark parameters. Effects of either 10-μM TIM or 4-mM GSH incubation of diabetic cardiomyocytes for 1 hour on the fluorescence changes related with Ca^2+^ sparks amplitude (A), sparks frequency (B), time to peak amplitude TP (left) and full duration half maximum, FDHM (right) (C), and full width of half maximum of the Ca^2+^ sparks (D). Bars represent control (CON; n_rat_ = 5, n_cell_ = 30, n_spark_ = 102), diabetic (DM; n_rat_ = 5, n_cell_ = 35, n_spark_ = 122) and TIM or GSH incubated (+TIM or +GSH) diabetic (n_rat_ = 6, n_cell_ = 38 or n_cell_ = 32, n_spark_ = 120 or n_spark_ = 104) groups, respectively. Values are expressed as mean ± SEM. Significant at ^*^p<0.05 *vs*. CON, ^†^p<0.05 *vs*. DM.

Of note, essentially similar results on Ca^2+^-spark intensity and frequency were obtained after 1 hour incubation with 10-µM timolol or 4-mM GSH of cardiomyocytes isolated from diabetic rats, although at these concentrations GSH appeared more potent than timolol (Fig. 6A–D, respectively).

### Timolol-treatment of diabetic rats affects biochemical properties of RyR2 macromolecular-complex

**Figure 7:**
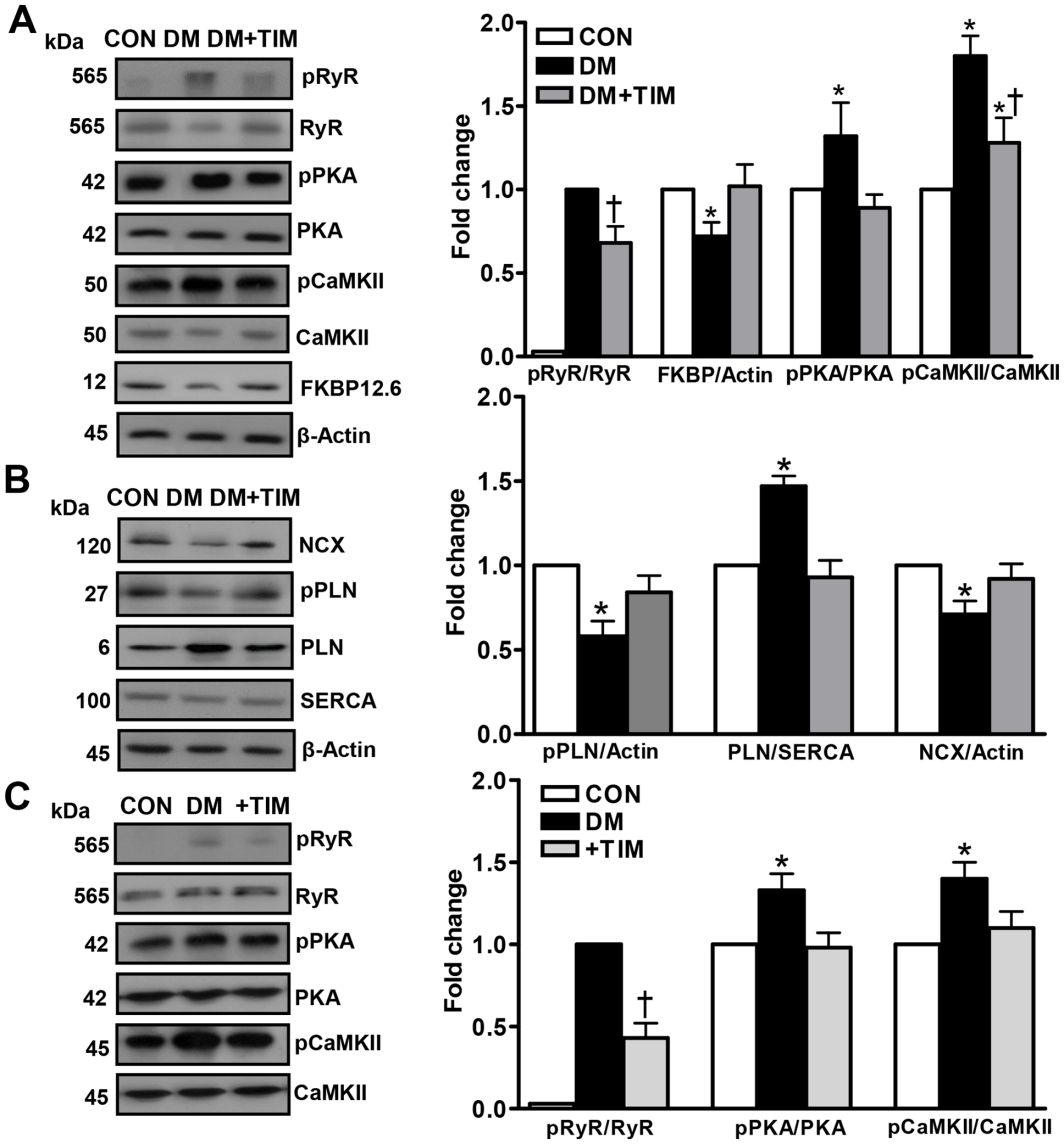
Effects of timolol on phosphorylation and protein levels of both SR Ca^2+^ release channel (RyR2) and some Ca^2+^ handling regulators. Data presented in (A) and (B) are obtained from homogenates from control (CON), diabetic (DM), and timolol treated diabetic (DM+TIM) rat hearts. (A) *Left*: representative Western blotting for 565 kDa phospho-RyR2-Ser^2808^ (pRyR2) and total RyR2, 42 kDa phospho-PKA-Thr198 (pPKA) and PKA, 50 kDa phospho-CaMKII-Thr286 (pCaMKII) and CaMKII, 12 kDA FKBP12.6, and 45 kDa β-actin, respectively. *Right*: quantification for the ratio of pRyR2 to RyR2, FKBP12.6 to β-actin pPKA to PKA, and pCaMKII to CaMKII, respectively. (B) *Left:* Western blotting for 120 kDa sarcolemmal NCX, 27 kDa phopho-PLN (Thr-17) and 6 kDa total PLN (L-15), 100 kDa SERCA2 (N-19), and 45 kDa β-actin, respectively. *Right*: quantification for the ratio of pPLN/actin, PLN/SERCA, and NCX/actin, respectively. The parameters given in section (C) are presented for ratio of 565 kDa pRyR2 to total RyR2, pPKA/PKA, and pCaMKII/CaMKII, respectively (right) in the isolated cardiomyocytes from the diabetic (DM) rats incubated with either 10-μM TIM (+TIM) or 4-mM glutathione, GSH (+GSH) (1 hour). *Left*: representative Western blotting for 565 kDa pRyR2 and total RyR2, 42 kDa pPKA (Thr198) and PKA, and 50 kDa pCaMKII-Thr286 and CaMKII, respectively. Bars represent mean ± SEM, n = 5–6 for hearts/group/protocol (double assays in each sample from each group for each type of measurement). Significant at ^*^p<0.05 *vs*. CON and ^†^p<0.05 *vs*. DM.

Under most pathological conditions including diabetes, alterations in the characteristics of intracellular basal free Ca^2+^ transients occur in combination with altered phosphorylated levels of RyR2 (pRyR2; 565 kDa phospho-RyR2-Ser^2808^). The pRyR2 level in heart homogenates of 6-month-old control, diabetic and timolol-treated diabetic rats was thus evaluated by using specific antibodies directed against RyR2 and its phosphorylated form. Similar to our previous studies [3], [51], diabetic group showed a significant decrease in protein level of RyR2 compared to the age-matched controls. Treatment with timolol reversed the depressed protein levels of RyR2 in the diabetic rats compared to the untreated diabetic rats (data not shown). Furthermore, in line with the previous studies, a marked hyperphosphorylation of RyR2 occurred in diabetic rats compared to the age-matched controls, while timolol-treatment of the diabetic rats significantly repressed this enhancement (Fig. 7A). The amount of FK506-binding protein (FKBP12.6) that stabilizes RyR2, thus preventing aberrant activation of the channel during the resting phase of the cardiac cycle, is decreased in the diabetic heart [3]. Similarly, timolol-treatment restored the FKBP12.6 protein level in diabetic rat heart (Fig. 7A).

The phospho-PKA level, pPKA (42 kDa phospho-PKA-Thr198), known to activate RyR2 and induce Ca^2+^ release, was estimated in the heart homogenates from three groups of animals. There was no difference in PKA protein level between the treated and the untreated diabetic groups (data not shown), while there is a significant increase in the pPKA level of untreated diabetic group that was prevented significantly with timolol treatment (Fig. 7A). There were no significant differences in the protein levels of CaMKII (an important protein regulating of intracellular Ca^2+^ level among the treated diabetics) untreated diabetics, and the controls while there was a significant increase in phospho-CaMKII, pCaMKII (50 kDa phospho-CaMKII-Thr286) level of the diabetic group compared to that of the control. Timolol-treatment also significantly prevented this increase.

### Effects of timolol-treatment of diabetic rats on total amount of SR Ca^2+^-ATPase (SERCA), sarcolemmal Na^+^/Ca^2+^-exchanger (NCX), phospholamban (PLN), and phospho-PLN

To test whether the depression in SR function was associated, in part, with any other Ca^2+^-handling regulators besides RyR2-macromolecular complex proteins, we analyzed the changes in total expression levels of SR Ca^2+^-ATPase (SERCA2; N-19 at 100 kDa) and total phospholamban (PLN; L-15 at 6 kDa), and the phosphorylated level of PLN (Thr-17 at 27 kDa) among the groups. Western blot analysis demonstrated that, compared with the control group, the level of PLN (129±10%) with respect to the β-actin and the ratio of PLN to SERCA (147±6%) were significantly different in the diabetic group and these were normalized significantly with timolol-treatment (Fig. 7B). On the other hand, either diabetes or timolol-treatment of diabetics did not affect significantly the SERCA levels (88±10% and 102±4%, respectively) with respect to that of the control. However, the phospho-PLN (relative to that of β-actin) in the diabetic group was found to be significantly depressed (50±9%) compared to that of the control, while it was normalized with timolol-treatment. Therefore, the calculated ratio of phospho-PLN to total PLN indicates a marked decrease in the diabetic group in comparison with the control group (39±9%), while again it was significantly prevented with timolol-treatment (73±13%).

The diabetic rats also displayed abnormal cardiac sarcolemmal Na^+^/Ca^2+^-exchanger (NCX1 at 120 kDa) protein level. NCX level was decreased significantly (71±12%) in the diabetic rats compared with that of the control, while timolol-treatment presented a significant preservation (92±11%) (Fig. 7B).

### Acute effect of timolol in hyperglycemic cardiomyocytes under *in vitro* conditions

To assess further the antioxidant effect of timolol on diabetic cardiomyocytes, diabetic cardiomyocytes were incubated with timolol and then the RyR2 phosphorylation level was measured. Incubation of diabetic cardiomyocytes with 10-μM timolol for 1 hour significantly prevented the hyperphosphorylation level of RyR2 without any significant effect of its protein level (Fig. 7C). Timolol also significantly reduced the phosphorylated states of PKA and CaMKII in diabetic cardiomyocytes without any significant effect of their protein levels (Fig. 7C).

### Timolol has antioxidant action in both circulatory system and myocardium of diabetic rats

**Figure 8:**
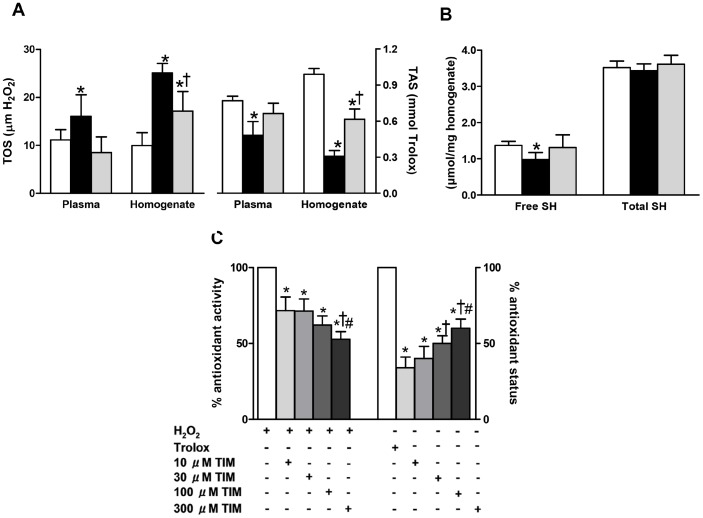
Confirmation of antioxidant effect of timolol on the contractile activity of diabetic rat heart via using different biochemical approaches. The total oxidant status measured with respect to H_2_O_2_ and the total antioxidant status measured with respect to trolox (A) in both plasma and heart homogenate of control rats (CON group; white bar) and diabetic rats without treatment (DM group; black bar) or with TIM treatment (DM+TIM group; gray bar) for 4-week following the one week of diabetic status confirmation. (B) The total and free protein thiol levels measured in heart homogenate of the groups. Bars represent mean ± SEM. The number of rats is 12–17 for rats/group/protocol. Significant at ^*^p<0.05 *vs*. CON group, ^†^p<0.05 *vs*. DM group. (C) *In vitro* antioxidant activity and antioxidant status of TIM was measured with colorimetric methods. Antioxidant activity of increasing concentration of TIM is evaluated with absorbance changes in H_2_O_2_-induced signal. Bar graphs represent mean ± SEM values for 10-30-100-300 μM TIM applications on H_2_O_2_ included samples for expressing antioxidant activity. Bar graphs represent mean ± SEM values from Trolox or 10-30-100-300 μM TIM applications on ABTS chromogen solution for expressing antioxidant status (triple assays in each sample for each type of measurement). Significant at ^*^p<0.05 *vs*. H_2_O_2_ or Trolox, and ^†^p<0.05 *vs*. 10-μM TIM, ^#^p *vs*. 30-μM TIM.

To be well-known circulating plasma markers of oxidative stress in STZ-diabetic rats, increased total oxidant status (TOS) and decreased total antioxidant status (TAS) measured in plasma were significantly antagonized after timolol treatment of diabetic rats (Fig. 8A). To further explore whether the increased oxidative stress and decreased antioxidative defence system in chronic diabetic rats can also induce similar changes in the myocardium, we also analyzed the TOS and TAS levels in the heart homogenates. The TOS level was higher, while the TAS level was found to be lower in untreated diabetic rat heart homogenates compared with those of the controls. Significantly, timolol-treatment of diabetic rats for a 12-week period prevented these changes (Fig. 8A). The relative contents of free and total protein thiols were also analyzed in the heart homogenates of these three groups of rats. Free protein thiol content was markedly decreased in untreated diabetics compared to age-matched controls, and was restored with timolol-treatment, while the total protein thiols were found to be similar in these three groups (Fig. 8B).

To assess further the antioxidant effect of timolol in diabetic rat heart, in part due to its action on some modification of sulfhydryl groups in diabetic heart, as presented previously by an early study [52], the relative levels of both free and total protein thiols were also analyzed in the heart homogenates. The free protein thiol content was markedly decreased in the untreated diabetic rats compared with the age-matched controls, and they were restored with timolol-treatment, whereas the total protein thiols were found to be similar among these three groups (Fig. 8B).

### Direct antioxidant action of timolol

To test whether the observed timolol effects in diabetic cardiomyocytes as well as diabetic rats are due to its putative scavenging activity for ROS (O_2_^−^, H_2_O_2_, OH^.^), we investigated its direct antioxidant effect in H_2_O_2_-induced oxidant medium (Fig. 8C). Timolol exerted a clear antioxidant effect in this fully oxidized medium in a concentration-dependent manner. Also dose-dependently, antioxidant status of timolol was increased, respect to Trolox (well known antioxidant) solution. We repeated similar experiments with another β-blocker propranolol. Propranolol, at any concentration (from 10 to 1500 µM), did not show any antioxidant effect in the presence of H_2_O_2,_ in the oxidized medium, as earlier reported by Gomes et al. [35] who suggested that timolol was more effective on ROS scavengers whereas an antioxidant role of propranolol was on the reactive nitrogen species (RNS) scavengers.

## Discussion

The present data, in male rats with 12-week diabetes following streptozotocin injection, showed that simultaneous chronic treatment with timolol, a β-AR blocker, markedly prevented diabetes-associated cardiac alteration as evidenced by preserved left ventricular function and cellular electrical activity. The main finding of this study is to demonstrate an association of timolol effect in diabetic rat heart with normalization of the sarcoplasmic reticulum (SR) Ca^2+^ release channel ryanodine receptors (RyR2) macromolecular complex together with normalization of intracellular free Ca^2+^ ([Ca^2+^]_i_) homeostasis, including also the [Zn^2+^]_i_ homeostasis. Our present study indicates that a treatment of the diabetic rats with a β-blocker, timolol prevented the depressed maximal contractile responses to isoproterenol, ISO stimulation slightly but significantly in a time-dependent manner similar to that of propranolol effect in the similar animal model [53], without affecting the total β-AR density. Our present data are in line with the previous data carried out with different β-blockers such as carvedilol, metroprolol, or atenolol, of which reduced left ventricular volume and improved cardiac function in diabetic rats [25], [26]. Beta-blockers are widely used for the treatment of cardiovascular and noncardiovascular diseases. Indeed, in even an early review article, it had been mentioned that severe heart failure due to idiopathic dilated cardiomyopathy could be improved in patients receiving beta-blocker therapy starting at a very low dose and followed by a stepwise increase depending on the treatment duration [54]. Nevertheless, although many patients were treated with metoprolol, carvedilol or bucindolol for periods of 2 to 12 months, their mechanism of action is not fully known. Furthermore, in here, timolol, itself demonstrates antioxidant activity and antagonizes the increased oxidative stress while some of its effects on [Ca^2+^]_i_ and [Zn^2+^]_i_ homeostasis were mimicked by reduced glutathione. Accordingly, we proposed that the beneficial effects of timolol-treatment on the cardiac activity in diabetic rats results, in most part, from its antioxidant properties.

Defective intracellular Ca^2+^ signaling contributes to cardiomyopathy in type 1 diabetic rats. In the previous studies, we and others have demonstrated that depression in both contraction and relaxation activities of Langendorff-perfused whole heart, reduced amplitude and prolonged time course of the Ca^2+^ transients, as well as increase in intracellular basal free Ca^2+^ level, are associated with anomalous Ca^2+^ pumping, Na^+^/Ca^2+^ exchange, and more recently to altered RyR2 behaviour as well as some Ca^2+^ handling regulators such as phospholamban (PLN) [3], [10], [11], [12], [13], [14]. In their study, Yaras et al. [3] reported that Ca^2+^-spark frequency of cardiomyocytes isolated from 5-week STZ-induced diabetic rats significantly increased with respect to aged-matched control rats while their amplitude is reduced. In this group of diabetic rats, spatio-temporal properties of the Ca^2+^ sparks were also significantly altered to being almost parallel to the changes of Ca^2+^ transients. In addition, RyR2 from diabetic rat hearts were hyperphosphorylated and protein levels of both RyR2 and calstabin2, FKBP12.6 depleted [3]. Here, our present data in cardiomyocytes from rats with 12-week diabetes reinforced the observations from 5-week diabetic rats. More importantly, in here, our data showed also a marked increase in the fluorescence ratio of basal [Zn^2+^]_i_ besides that of the [Ca^2+^]_i_. Notably, it has been previously shown that exogenously applied oxidants caused about a 30-fold increase in the resting level of [Zn^2+^]_i_ but only doubled in the [Ca^2+^]_i_ in freshly isolated cardiomyocytes [18]. Furthermore, previous reports have shown that the basal free [Zn^2+^]_i_ is increased by 70% in cardiomyocytes from male diabetic rats being parallel to an unbalanced oxidant-status/antioxidant capacity in the same heart preparations [20], and by over 200% in aldosteronism [55]. Coordinated changes in the basal [Ca^2+^]_i_ and [Zn^2+^]_i_ levels have recently been reported under experimental conditions (intracellular Zn^2+^ overload or intracellular Ca^2+^ decrease), as well as during a single beat (transients or elementary events) [28].

Zinc ion and redox status demonstrate a complex interplay in cells. The [Zn^2+^]_i_ level is modulated by the redox state and the level of reactive oxygen species, ROS in cells [56] while Zn^2+^ increases the antioxidant capacity of the cells or leads to the release of toxic ROS [57]. Indeed, the [Zn^2+^]_i_ distribution is linked to redox metabolism despite Zn^2+^ itself not being redox active and generally Zn^2+^-proteins being redox-inert. Therefore, it has been long viewed as a component of the antioxidant network, and growing evidence points to its involvement in redox-regulated signaling. Zinc-coordination environments with cysteine ligands have the remarkable property that the sulfur-ligands can be oxidized and then reduced with concomitant release and binding of Zn^2+^ [57]. About 30% of the Zn^2+^-buffering capacity emanates from sulfur donors (thiols) and thus serves as redox (oxidant) buffer capacity at the same time [56]. Moreover, Zn^2+^ elevates ROS, in living cells by inhibiting mitochondria [58] and activating NADPH oxidase [59] although it has been shown in an early study that oxidative phosphorylation rate and Mg^2+^-dependent ATPase activities were depressed in mitochondria from left ventricle of diabetic rat heart contributing into development of cardiomyopathy at late stages of diabetes [60]. However, in a recent work, it has been shown that both extra-and intracellular Zn^2+^ modulates L-type Ca^2+^ channel properties, as well as its regulation by β-adrenergic agonists independently of altering the cellular redox status [61]. The reduced Ca^2+^-current density that we observed in diabetic cardiomyocytes (Fig. 2D) might be attributable to the alteration in the intracellular basal free Zn^2+^, despite the fact that we were using the whole-cell perforated patch-clamp in which the cytolosic medium is in most part substituted by the intracellular pipette solution. In addition, our present work demonstrated, for the first time, that an increase in [Zn^2+^]_i_ is associated with hyperphosphorylation of the RyR2 in a concentration-dependent manner, at most via phosphorylation of both PKA and CamKII under acute zinc-inophore ZnPT exposure rather than a direct hyperphosphorylaion of RyR2 with increased [Zn^2+^]_i_. This hypothesis depending on our observations is further supported with the data on multiple functional effects of [Zn^2+^]_i_ on CaMKII and modulation of RyR1 binding to SR vesicles in skeletal muscle biphasically [48], [49]. Indeed, it is well-accepted that [Zn^2+^]_i_ level plays critical roles in the redox signaling pathway and maintaining the normal structure and physiology of various cell types [62]. Therefore, it can be hypothetized that Zn^2+^ may compete with or substitute for metal ions crucial for the activity of signaling proteins. Accordingly, supporting this last statement, Zn^2+^ is also known to induce CaMKII autophosphorylation, inhibit protein tyrosine phosphatases [48] while divalent metal ions influence catalysis and active-site accessibility in the cAMP-dependent protein kinase [50]. In here, although a likely role for such [Zn^2+^]_i_ signals is the modulation of protein phosphorylation, it is a strong possibility that both activated/phosphorylated CaMKII and PKA due to increased [Zn^2+^]_i_, turn into a hyperphosphorylated RyR2 under high extracellular Zn^2+^ exposure. Therefore, increased [Zn^2+^]_i_, most probably a contribution of increased [Ca^2+^]_i_, under increased oxidative stress together with depressed antioxidant-defence in the cells via hyperglycemia induces important defects in excitation-contraction coupling of cardiomyocytes. The Zn^2+^ **-**dependent RyR2 hyperphosphorylation thereby correlates a possible indirect contribution of increased level of [Zn^2+^]_i_ into RyR2-dependent dysregulation of diastolic Ca^2+^ in diabetic cardiomyocytes. There are several Zn^2+^ signaling pathways involved in intracellular Zn^2+^ homeostasis. Possibly, Zn^2+^ disbalance results in a signaling disbalance caused by a local surplus of Zn^2+^ interfering with cellular signaling networks. Therefore, it can be clearly seen that the [Zn^2+^]_i_ signaling can easily interfere with the the [Ca^2+^]_i_ signaling of cardiomyocytes, particularly under pathological conditions, underlying, in part, cardiac dysfunction.

Therapeutic effects of β-adrenergic antagonists are generally explained by their capacity to block β-adrenoceptors (β-ARs), and β_1_-AR blockage has become the chosen therapy in the treatment of congestive heart failure [63]. It is one of general textbook information that any major injury that reduces cardiac pumping capacity activates neurohumoral mechanisms, including the sympathetic system. Under pathophysiological conditions, acute increase in norepinephrine serves as a fast and powerful mechanism that stabilizes perfusion of crucial organs by redirecting blood flow away from muscles, kidney, etc., and stimulating heart rate and developed force. However in pathological situations such as hyperglycemia or diabetes, if the primary pumping defect of the heart continues, the sustained increase in sympathetic tone turns from friend to foe, primarily for two reasons: first, peripheral vasoconstriction and increased load lead to reduced cardiac output; second, sustained stimulation of cardiomyocytes leads to an overproportional increase in cell death, fibrosis, and hypertrophy in cardiomyocytes. Collectively, these changes accelerate the decline of the heart function. In heart failure, the signaling cascade is normally desensitized in response to chronic norepinephrine stimulation. The diabetic heart is more susceptible to hypertensive or ischemic injury as a result of a number of pathological changes including cell death, increased oxidative stress, impaired Ca^2+^ handling, and alterations in second messenger signaling pathways. As summarized in the introduction, some of the beneficial cardiovascular effects of β-ARs blockage have already been shown to be associated with their antioxidant properties by scavenging endogenous oxidants [30], [31]. Indeed, oxidative stress plays several critical roles in the development of diabetic cardiovascular complications, including myocardial hypertrophy. Diabetes-induced heart dysfunction is associated with a very low plasma insulin level, and an unbalanced level of oxidative stress to antioxidant defense ratio, while oxidative stress in diabetic animals is reversed with antioxidants [20], [64]. To date, molecular mechanisms underlying the dysregulation of SR-Ca^2+^ release channel ryanodine receptors (RyR2) during chronic diabetes remains incompletely understood. However, alterations in the sensitivity of RyR2 to Ca^2+^ activation could result from oxidation of RyR2 by ROS and/or reactive carbonyl species (RCS) [17], [65], besides increases in phosphorylation by PKA at Ser^2808^ and by CaMKII at Ser^2808^ and Ser^2814^ sites [66]. In the present study, long-term timolol treatment maintains normal intracellular Ca^2+^ signaling in cardiomyocytes via preventing RyR2 hyperphosphorylation in STZ-injected rat hearts. A similar preserved channel regulation in RyR2 with improved cardiac function was previously shown by Doi *et* *al.* in tachycardia-induced canine heart failure [25] and by Tuncay *et* *al.* with propranolol treatment of diabetic rats [28]. In these studies, propranolol had no effect on the protein level of SERCA as opposed to a previous report describing an enhancement of SERCA expression and activity with β-blockers in human heart failure [67]. This discrepancy might be attributed to the different experimental dose of β-blockers used or a different regulation of β-ARs antagonism. Beta-blockers are widely used for the treatment of cardiovascular and non-cardiovascular diseases. Nevertheless, their mechanism of action is not fully known, and is significantly different from other agents in this class. Nonselective β-blockers such as propranolol, metoprolol, or carvedilol can exert adrenoceptor-independent effects including scavenging of free radicals and inhibition of PKC leading to controlled cellular redox status and consequently functional recovery in organs including the heart [36], [68]. Indeed, a direct radical scavenging effect of the β-blockers has also been previously [36]. In a cell-line study, Miyamoto et al. [34] put forward that both nipradilol and timolol maintained potent protective action against increased oxidative stress. In other studies carvedilol demonstrated a marked beneficial effect in heart failure via scavenging free radicals, preventing Ca^2+^ leak due to stabilization of RyR2 in hearts with a marked failure [68]. Further, in a recent study, we demonstrated a beneficial action with the chronic timolol treatment on age-related alterations in heart function of 12-month-old female rats via regulation of cellular redox status [32].

Cardiac dysfunction in diabetes arises from a number of alterations in Ca^2+^ handling in cardiomyocytes. These usually lead to depressed cardiac Ca^2+^ transients and to decreased contractile function. Typically, these defects in Ca^2+^ handling include depressed SERCA function and/or increased NCX activity [10], [11], [13], [14] that lead to decreased SR [Ca^2+^]. In addition, cardiomyocytes from diabetic hearts typically exhibit an increase in the diastolic leak of Ca^2+^ from the SR [3]. This increase is also primarily due to increased RyR2 hyperphosphorylation, while a possible contribution of NCX activity cannot be excluded. Indeed in diabetic hearts, associated with a decreased RyR2 protein level, there is a marked reduction of NCX protein. The latter, despite a reduced influx of Ca^2+^ ions through the L-Type Ca^2+^ channels may contribute to diastolic Ca^2+^ overload since total NCX activity should be reduced as there is no change in [Na^+^]_i_ of diabetic cardiomyocytes [69].

Besides, compromised Ca^2+^ removal, one of the hallmarks of cardiac dysfunction including in diabetic cardiomyopathy [11], [13], [14] depends upon SERCA activity. Our classical knowledge emphasizes the importance of the fact that the amount of Ca^2+^ stored in SR depends, in part, on its balancing action, restoring the Ca^2+^ released during each cycle. SERCA is able to modulate this uptake under changing circumstances because it is regulated via an inhibitory protein, PLN which can be phosphorylated by both PKA and CaMKII at different sites of PLN [70], [71]. The molar ratio of PLN to SERCA can be conceived as a critical determinant of the contractile force and its regulation. This fact is also tested in this study: Diabetes induced a markedly increased phosphorylation level of PLN with unchanged levels of both PLN and SERCA, which are in line partially but not fully with previously published data [11], [13], [14]. The decreased PLN phosphorylation is consistent with not only the decreased function of SR but also with the absence of a hyperadrenergic state that is indicated by the slower heart rate in diabetic rats data [13]. Moreover, an early report by Kadambi et al. on targeted overexpression of PLN protein could cause cardiac contractile dysfunction is further supporting our present results [72].

Intracellular concentrations of redox-active molecules can significantly increase in the heart as a result of activation of specific signal transduction pathways or the development of certain pathophysiological conditions. Changes in the intracellular redox environment can affect many cellular processes, including the gating properties of ion channels and the activity of ion transporters [73]. Redox-mediated alterations of [Zn^2+^]_i_ and [Ca^2+^]_i_ homeostasis is directly involved in cardiac pathologies such as diabetes [18], [57]. Therefore, it can be suggested that redox modulation of biochemical pathways and signaling cascades related with either [Ca^2+^]_i_, [Zn^2+^]_i_, or both with any antioxidant can extend to normalize cardiac dysfunctions and other pathologies in diabetic subjects. On top of the marked antioxidant properties of timolol to scavenge ROS that might account, for the most part, for the prevention of the cardiac alterations in STZ-induced diabetic rats without affecting hyperglycemia, the regulation of [Zn^2+^]_i_ should have a major importance despite of the fact that elevated [Zn^2+^]_i_ might be helpful in offering protection from redox unbalance. In an early report from a study performed on a rodent heart with hereditary muscular dystrophy, [Ca^2+^]_i_ overloading and oxidative stress are also accompanied by increased [Zn^2+^]_i_ [74]. Besides, Kamalov et al [55] suggested that an optimal range of [Zn^2+^]_i_/[Ca^2+^]_i_ ratio in cardiomyocytes and mitochondria must be preserved to combat oxidative stress. Thus, the increased cytosolic and mitochondrial free Ca^2+^ level are coupled to the induction of oxidative stress, while antioxidant effects result from the rise in cytosolic and mitochondrial [Zn^2+^] levels accompanied by a simultaneous activation of metal response element transcription factor-1, and its induction of such antioxidants as metallothionein-1 and glutathione peroxidase. However, with a long-term treatment, by maintaining both a cellular redox-status and [Zn^2+^]_i_ level near to control level, timolol prevents the subsequent alterations in [Ca^2+^]_i_ homeostasis including Ca^2+^ release by RyR2, which leads to major defects in cardiac activity.
